# Melioidosis in Vietnam: Recently Improved Recognition but still an Uncertain Disease Burden after Almost a Century of Reporting

**DOI:** 10.3390/tropicalmed3020039

**Published:** 2018-04-09

**Authors:** Trung T. Trinh, Linh D. N. Nguyen, Trung V. Nguyen, Chuong X. Tran, An V. Le, Hao V. Nguyen, Karoline Assig, Sabine Lichtenegger, Gabriel E. Wagner, Cuong D. Do, Ivo Steinmetz

**Affiliations:** 1 Institute of Microbiology and Biotechnology, Vietnam National University, Hanoi 100000, Vietnam; 2 Department of Academic Affairs, Phan Chau Trinh University, Quang Nam 560000, Vietnam; nguyendongoclinh@gmail.com; 3 Department of Medical Microbiology, Hanoi Medical University, Hanoi 100000, Vietnam; nguyen.vu.trung@gmail.com; 4 National Hospital for Tropical Diseases, Hanoi 100000, Vietnam; 5 Department of Infectious Diseases, Hue University of Medicine and Pharmacy, Hue 530000, Vietnam; xuanchuonghue@gmail.com; 6 Department of Medical Microbiology, Hue University of Medicine and Pharmacy, Hue 530000, Vietnam; levanan.hump@gmail.com; 7 Department of Infectious Diseases, University of Medicine and Pharmacy, Ho Chi Minh 700000, Vietnam; haodiep61@gmail.com; 8 Hospital for Tropical Diseases, Ho Chi Minh 700000, Vietnam; 9 Institute of Hygiene, Microbiology and Environmental Medicine, Medical University of Graz, 8010 Graz, Austria; karoline.assig@medunigraz.at (K.A.); sabine.lichtenegger@medunigraz.at (S.L.); gabriel.wagner-lichtenegger@medunigraz.at (G.E.W.); ivo.steinmetz@medunigraz.at (I.S.); 10 Department of Infectious Diseases, Bach Mai Hospital, Hanoi 100000, Vietnam; doduy.cuong@gmail.com; 11 Friedrich Loeffler Institute of Medical Microbiology, University Medicine Greifswald, 17475 Greifswald, Germany

**Keywords:** melioidosis, *Burkholderia pseudomallei*, Vietnam, public awareness, animal, environment

## Abstract

The first cases of human melioidosis were described in Vietnam in the 1920s, almost a century ago. It was in Vietnam in the thirties that the saprophytic nature of *B. pseudomallei* was first recognized. Although a significant number of French and U.S. soldiers acquired the disease during the Vietnam wars, indigenous cases in the Vietnamese population were only sporadically reported over many decades. After reunification in 1975, only two retrospective studies reported relatively small numbers of indigenous cases from single tertiary care hospitals located in the biggest cities in the South and the North, respectively. Studies from provincial hospitals throughout the country were missing until the Research Network on Melioidosis and *Burkholderia pseudomallei* (RENOMAB) project started in 2014. From then on seminars, workshops, and national scientific conferences on melioidosis have been conducted to raise awareness among physicians and clinical laboratory staff. This led to the recognition of a significant number of cases in at least 36 hospitals in 26 provinces and cities throughout Vietnam. Although a widespread distribution of melioidosis has now been documented, there are still challenges to understand the true epidemiology of the disease. Establishment of national guidelines for diagnosis, management, and reporting of the disease together with more investigations on animal melioidosis, genomic diversity of *B. pseudomallei* and its environmental distribution are required.

## 1. Introduction

Although sporadic cases of melioidosis have been reported from Vietnam since the year 1927, the disease has only recently attracted the deserved attention among Vietnamese health care professionals. This neglect is particularly remarkable, given the fact that a fundamental characteristic of *B. pseudomallei*—namely, the environmental reservoir of this pathogen—was demonstrated for the first time in Vietnam [1,2]. Although many melioidosis cases among French and American soldiers were reported during the long-lasting armed conflicts, this had no sustainable effect on the recognition of melioidosis in the indigenous Vietnamese population. In this report, we summarize the history of melioidosis in Vietnam in humans and animals starting almost a century ago and finally describe more recent activities trying to unravel the burden of disease and to increase awareness. We also discuss current knowledge on environmental *B. pseudomallei* in Vietnam, the population structure of Vietnamese *B. pseudomallei* and its phylogenetic relatedness. Finally, we address current and future challenges in prevention and diagnosis of the disease.

## 2. Review of Melioidosis in Vietnam

### 2.1. Human Melioidosis

#### 2.1.1. 1920s to 1950s

The first case of human melioidosis in Vietnam was detected in 1925 in an ill-nourished female patient, pregnant in the fifth month, living at Thu Duc, close to Saigon (now Ho Chi Minh City). Six days after onset of symptoms, the patient had a miscarriage and, after 14 days of illness, the patient died [3]. *B. pseudomallei* was isolated from the blood at the Pasteur Institute of Ho Chi Minh City and the identification was confirmed at the Institute for Medical Research, Malaysia [4]. 

Subsequently cases were reported by Menard in 1928 from Ho Chi Minh City and Tonkin (now Hanoi in the North), as cited by Pons (1930) [5]. Further culture-confirmed cases of melioidosis, including cases related to traffic accidents (see below Section 3) were then reported at Hue city (Central Vietnam) and from the North [1]. In 1947, 28 cases of acute, sub-acute, and chronic melioidosis were described from various hospitals in the South [6]. Of those, only nine cases were Vietnamese patients. The others were 15 Caucasians, 2 black patients, and 1 patient of Chinese and 1 of south Asian descent. Fatal outcomes were reported in 19 out of those 28 cases, although the outcome was not documented for all patients. From 1951 to 1953, five pulmonary cases of melioidosis were diagnosed at hospitals in both southern Vietnam and France. All of the cases were French citizens with a history of residence or station in the region of Vietnam, Laos, and Cambodia [7]. A case of chronic pulmonary melioidosis was also described in a soldier suffering from a chest wound caused by a bullet in Vietnam [8]. Between 1948 and 1954, approximately 100 cases were reported among 400,000 French forces stationed in Vietnam, Laos, and Cambodia, as cited by Sanford (1978) [9]. In 1956, a fatal case of septicemic melioidosis was diagnosed in a 40-year-old south Vietnamese soldier admitted to Cong Hoa Hospital [10].

#### 2.1.2. 1960s to 1980s

It is interesting to note that already in 1967, Mo and Duong stated that the disease was most likely underdiagnosed in hospitals of southern Vietnam and that cases of melioidosis were probably misdiagnosed as tuberculosis or disseminated fungus infections. The authors also mentioned several cases detected annually in different hospitals of the South and described the development of melioidosis in a young soldier after a military operation in a swampy area of the South [11]. With the deployment of the U.S. Armed Forces in Vietnam, melioidosis started to be detected in the U.S. military personnel. As cited by Diamond and Pastore (1967), through February 1967, 35 cases were diagnosed in U.S. troops stationed in Southeast Asia, with 8 fatal cases reported [12]. Those patients were diagnosed because diseased or wounded soldiers were referred to modern hospitals of either Republic of South Vietnam or United States, where microbiological laboratories were available for diagnosis of melioidosis as well as for other infectious diseases. Within the year of 1966, a series of 9 cases of pulmonary melioidosis in U.S. soldiers was described [13]. Another nine cases of melioidosis in U.S. soldiers, including four fatal cases, were diagnosed in Vietnam in Long Binh (now Dong Nai province in the South) [14]. Since more cases of melioidosis were subsequently reported in U.S. soldiers, melioidosis became one of six major tropical infectious diseases for which U.S. physicians needed to maintain a high index of suspicion in febrile soldiers returning from Vietnam [15]. From April 1965 to December 1969, 187 cases with 13 deaths were reported in the U.S. Army personnel stationed in Vietnam. Until the U.S. withdrawal in 1972, approximately two to three culture-confirmed cases were detected among the U.S. soldiers every month [9,16,17]. Serology using indirect hemagglutination (IHA) showed that 8.9% of the sera from the Vietnam veterans had titers of 1:40 or greater [18]. Based on such titers it was estimated by Clayton et al. that approximately 250,000 among three million U.S. Army personnel got infected with *B. pseudomallei* when serving in Vietnam [18,19]. Although these estimates might be interpreted with some cautions considering the limited specificity and sensitivity of the non-standardized IHA test, this study indicated a potential reservoir of latent *B. pseudomallei* infection among personnel returning from Vietnam. Indeed, it became obvious that latent infection and reactivation, sometimes after many years, does occur [20]. This phenomenon led to the melioidosis nickname ‘Vietnam Time Bomb’ [21]. A possible human-to-human transmission via sexual contact was suggested in the wife of a returning Vietnam veteran with prostatitis due to *B. pseudomallei* infection [22]. Melioidosis was also diagnosed in a newborn whose father had served in Vietnam, although the source of a possible transmission remained unclear [23]. Despite this large number of melioidosis cases related to Vietnam during this period of time, very little information on the disease was reported for the indigenous population (Table 1).

#### 2.1.3. 1990s until Present

In 1991, a medical thesis defended at Hanoi Medical University described 16 cases detected in hospitals of Hanoi over a 10-year period from 1980 to 1990, with seven fatal outcomes [29]. A study by Phung et al. (1993) using an IHA test revealed seropositivity in populations living in suburban communities of Hanoi ranging from 6.4% to 31.8%. This study also observed an association of seropositivity with rice farming [30]. In a study on cellular lipid and fatty acid composition of *B. pseudomallei*, seven patients with melioidosis were listed from whom strains were isolated in Vietnam between 1981 and 1991 [24]. In 1999, a retrospective study in the South reported only nine culture-confirmed cases with melioidosis from 3653 blood cultures of febrile patients admitted to the largest hospital for tropical diseases in Ho Chi Minh City from 1992 to 1998 [26]. Antibiotic treatment and patient outcomes were not reported in this study. In 2008, another retrospective study in the largest general hospital in the North reported 55 culture-confirmed cases observed in a period of time from 1997 to 2005. Analysis of clinical data showed that septicemia with pneumonia was the most common clinical presentation and diabetes was the most common risk factor. Seventeen out of 40 septicemic patients died, with nine deaths occurring within 48 h after admission. Based on the residential addresses of the patients, it was concluded that melioidosis is widely distributed and occurs in at least 18 of 25 northern provinces [27]. In the context of the Research Network on Melioidosis and *Burkholderia pseudomallei* (RENOMAB; see below Section 5) that started in 2014, a recent study reported 70 cases detected within seven months at five hospitals in North Central Vietnam. During the study period, the detection rate of *B. pseudomallei* ranged from 3.4% to 10.2% among positive blood cultures in those hospitals. Fifty-eight patients had septicemia. Of the 36 patients with known outcome, 18 patients died, with 6 deaths occurring within 48 h after admission [28]. However, as for other regions, the true burden of melioidosis in North Central Vietnam still needs to be determined (see below Section 7). The few epidemiological data available for Vietnam imply that rice farmers are at particular risk to acquire a *B. pseudomallei* infection [26,27,28].

### 2.2. Animal Melioidosis

#### 2.2.1. 1930s to 1960s

At the time when the first human case of melioidosis was detected in Vietnam, the disease was considered to be a zoonosis and animals a reservoir for *B. pseudomallei*. This assumption was based on the observation that *B. pseudomallei* could infect laboratory rodents and its virulence was comparable to *Yesinia pestis*, the causative agent of plague. However, a large microbiological study of more than 20,500 rats collected in southern Vietnam found only one rat to be culture-positive for *B. pseudomallei* as cited by Luong (1956) [31]. None of the 560 wild rats caught in Hanoi were positive for *B. pseudomallei* by culture [1]. Serological studies showed that serum collected from pigs contained antibodies against *B. pseudomallei*, as cited by Luong (1956) [31]. In 1954, melioidosis was detected in rabbits and guinea pigs at the Pasteur Institute of Ho Chi Minh City, as cited by Luong (1961) [32]. In 1955, Luong described culture-confirmed melioidosis cases in pigs at a pig farm near a rubber plantation in Thu Dau Mot (now Ho Chi Minh City), with the isolation of *B. pseudomallei* from creamy pus in lung and spleen abscesses [31]. Using serology and culture methods, several deaths of pigs were confirmed to be caused by melioidosis in the farms at Tan Son Nhat and Gia Dinh (now both are Ho Chi Minh City) [32].

#### 2.2.2. 1970s until Present

In 1971, cases of melioidosis were reported in the U.S. Army dogs in the Republic of South Vietnam. During a six-month period, 31 working dogs died, with four confirmed melioidosis cases. *B. pseudomallei* was cultured from the dogs’ lungs and various organs. The dogs came from different units in diverse locations in Vietnam. All of the dogs showed lesions in the lungs, epididymides, and testes [33]. The author stated that melioidosis remained underdiagnosed because bacteriologic cultures were not routinely performed at necropsy of the U.S. Army working dogs and that the disease does not seem to be uncommon in dogs. A serologic surveillance on 64 healthy U.S. military scout and tracker dogs after service in the Republic of South Vietnam showed that 12 (19%) dogs had developed antibodies against *B. pseudomallei*, with IHA titers higher than 1:80 [34]. After reunification in 1975, no further information about melioidosis in pigs or other livestock, as well as dogs, has been reported in Vietnam. Only recently, in the context of RENOMAB, a study carried out by a group at the National Institute of Veterinary Research reported a culture-confirmed melioidosis case of a pig in a farm at Nghe An province in North Central Vietnam [35].

## 3. *B. pseudomallei* in the Environment

The first experimental indication of the saprophytic nature of *B. pseudomallei* and its environmental reservoir was provided in Vietnam by Vaucel in 1937 [1]. His studies were triggered by the observation that individuals who developed melioidosis after traffic accidents were either immersed for a prolonged time in water of a pond or had a skull wound contaminated with mud. To test the hypothesis that *B. pseudomallei* exists in the environment, Vaucel submerged the scratched abdomen of guinea pigs into water of a pond collected in the North. Five days later, a moribund animal was sacrificed and *B. pseudomallei* was isolated from pus of the liver and spleen and other sites on solid media in pure culture [1]. About 20 years later in 1955, Chambon provided final evidence by directly isolating seven *B. pseudomallei* strains from five environmental samples including pond muds, rice field water, and a sample of pond water collected in the South [2]. In 1961, Luong provided further evidence for environmental *B. pseudomallei,* when two strains were cultured from water samples of a water spinach plantation from southern Vietnam [32].

Apart from this early environmental work, there are only a few more recent studies sampling rice fields for *B. pseudomallei*. In 1991, a study reported the isolation of *B. pseudomallei* from 4 out of 240 soil samples, and 1 out of 190 surface water samples collected in rice fields at four communities surrounding Hanoi was positive for *B. pseudomallei* [30]. Between 1992 and 1998 soil samples were collected from 137 rice fields around Ho Chi Minh City in southern Vietnam and nine fields were found to be positive [26]. The low *B. pseudomallei* detection rate from soil in those sampling studies is likely to be the result of the current culture protocols for environmental *B. pseudomallei* which have a limited sensitivity. It has been shown recently, that a multitarget quantitative PCR approach improves the detection rate and can predict cultivability of *B. pseudomallei* [36]. By using this multitarget qPCR, *B. pseudomallei* was detected in 35 (83.3%) out of 42 soil samples collected at 28 rice fields in southern Vietnam. From those samples, *B. pseudomallei* strains could be isolated from six (14.3%) samples by using conventional culture methods [36].

A recent prediction of the global environmental presence of *B. pseudomallei* at 5 × 5 km^2^ spatial resolution suggested that predominant parts of Vietnam are highly suitable for the environmental occurrence of *B. pseudomallei* [37]. However, mountainous areas near the border with Laos and China, especially in the Northwest region, some regions in the Central Highlands, and a zone involving the lower South Central Coast were found to be less suitable. It will be most important to test these predictions in future environmental studies and also to investigate the environmental factors involved in creating a habitat for *B. pseudomallei* at a high spatial resolution. The country harbors an enormous diversity of habitats ranging from tropical rain forest to dry forest, natural grassland, and agricultural land such as rice paddies to wetland habitats including rivers and lakes and coastal wetlands. Apart from the limited studies on rice fields, there is no information on the potential role of such different habitats as environmental reservoirs for *B. pseudomallei*. Since Vietnam has an enormous north–south expansion, including humid subtropical climate in the north, tropical monsoon climate in the center, and tropical wet and dry climate in the south, the potential influence of climate factors on the environmental presence of *B. pseudomallei* needs to be investigated. For the development of any preventive strategies or environmental countermeasures it will be important to define risk areas for infection and the role of different habitats and climate factors more precisely.

## 4. Phenotype, Genomic Diversity, and Phylogenetic Relatedness of *B. pseudomallei*

When the first *B. pseudomallei* strain was isolated from clinical specimens in 1925, the occurrence of different morphotypes, in this case rugose and ultra-rugose colonies on agar, were described [4]. As we now know various morphotypes represent a frequent characteristic of this species. Subsequent work demonstrated virulence of clinical and environmental *B. pseudomallei* strains in experimental infections and described antibiotic susceptibility to drugs available at that time [4,8,11,38]. A more recent study with 25 strains from northern Vietnam reported an antibiotic resistance profile that is also found in other endemic areas, with susceptibility to currently-recommended antibiotics for treatment of melioidosis such as ceftazidime, imipenem, meropenem, and co-trimoxazole. Resistance and intermediate resistance to tetracycline were noted for one strain and three strains, respectively [27].

In a study describing physiological and biochemical characteristics of 15 environmental and clinical *B. pseudomallei* strains from northern Vietnam, all strains shared typical *B. pseudomallei* characteristics with respect to motility, salt tolerance, growth temperature, sugar assimilation, cytochrome c oxidase and acid production, and colony morphology on different routine agar media [24]. The composition of cellular lipids and fatty acids were similar among the tested strains [24]. Biochemical and antigenic characteristics typical for *B. pseudomallei* were also described by Phuong et al. (2008) using the API 20 NE system and through agglutination of those strains with a *B. pseudomallei*-specific monoclonal antibody [27].

The *Burkholderia pseudomallei* ‘Multi-Locus Sequence Typing’ (MLST) database is a rich resource to assess the genomic population structure based on seven housekeeping genes (https://pubmlst.org/bpseudomallei/ [39]. Since the development of the *B. pseudomallei* MLST scheme in 2003 [40], 104 isolates from Vietnam have been deposited in the database (5309 isolates overall) as of 14 February 2018 [27,41]. A total of 61 sequence types (STs) were identified, emphasizing the great genotypic diversity of the sampled population in this region. Of those, 31 STs were uniquely reported in Vietnam so far and most of the remaining STs are shared with neighboring countries like Thailand or Cambodia. The co-occurrence of the latter and the concomitant lack of regional specificity have already been noticed for other STs [41]. As already implied by Phuong et al. (2008), analysis of current *B. pseudomallei* MLST data showed the Vietnamese strains to mainly cluster with other Asian isolates as opposed to the Australian STs.

However, special care must be taken when analyzing *B. pseudomallei* MLST data. Its high recombination rate complicates the inferences of phylogenetic relationships of sequence types (STs) [42,43]. Nevertheless, the unprecedented amount of deposited data and the comparatively cheap prices render it a valuable tool, despite the emergence of next generation sequencing. With the advent of next generation sequencing (NGS), whole genome sequencing (WGS) has proven a powerful tool to study population genetics at a much higher resolution. This was recently shown by De Smet et al. (2015), reporting two strains, which shared one ST due to homoplasy, but could be distinguished by WGS [44].

Phylogentic studies based on single nucleotide polymorphisms (SNP) present strong evidence for an Australian *B. pseudomallei* origin [43,45] and a single transmission event to Southeast Asia [43,46]. In a recent study by Chewapreecha et al. (2017) on the global evolution and prevalence of *B. pseudomallei*, 19 strains from Vietnam (spanning a time period from 1947 to 2011) were subjected to whole genome sequencing and placed in a global context (469 isolates) [46]. A SNP-based phylogenetic analysis of these genomes clearly resolved Australian, Asian, and African/American clusters.

Strains from Vietnam appeared in 7 of 17 Asian subgroups, with most of the other members of the subgroup being from Thailand and Cambodia and belonging to the ‘Mekong sub-region’. The other Asian subgroups contained mainly isolates from Malaysia and Singapore (‘Malay cluster’). It could be shown that the transitions of *B. pseudomallei* between these two sub-regions was less than within the sub-region. As noted by the authors, this observation might be linked to trading networks and cultural links. Furthermore, the results indicate that the Mekong sub-region might have been a hotspot for the evolution of *B. pseudomallei* in Southeast Asia. Unfortunately, in contrast to other Asian clusters, no time could be estimated for the emergence of the most recent common ancestor of the Vietnam-containing subgroups. Besides, the study also demonstrates the potential of WGS to account for geographical differences in disease outcome, by screening for region-specific (virulence) loci [46]. The insights, based on just 19 Vietnamese isolates, already imply the wealth of information that can presumably be gained from such a high level of resolution. Should more rigorous clinical and environmental sampling schemes be applied and a higher number of strains analyzed, this will likely increase our knowledge of *B. pseudomallei* phylogeny and virulence tremendously. There is no doubt that these are interesting times for studying the genomic diversity of *B. pseudomallei* in Vietnam.

## 5. Activities to Raise Awareness of Melioidosis in Vietnam and Major Achievements

As in many parts of the world, neither melioidosis nor characteristics of *B. pseudomallei* are mandatory components of curricula at medical universities in Vietnam. This leads to a lack of knowledge among many doctors and other health care professionals about how to diagnose and manage the disease. As outlined above, this limited clinical awareness together with limited laboratory resources result in significant underdiagnosis. This has been very obvious in the central part of Vietnam. Although it is located in the same geographical belt and is close to highly endemic areas of Laos and northeast Thailand, cases of melioidosis were not reported from there until recently.

In 2014, a bilateral project called Research Network on Melioidosis and *Burkholderia pseudomallei* (RENOMAB), sponsored by the German Ministry for Education and Research and the Vietnamese Ministry of Science and Technology, was started. The project involved 40 national and regional hospitals in 27 provinces and cities throughout the country. By organizing a series of workshops on diagnosis of melioidosis for laboratory staff, hundreds of culture-confirmed cases have recently been detected [28]. Two national scientific conferences on melioidosis were organized in order to raise awareness of the disease among infectious disease physicians, microbiological laboratory staff, medical teachers, researchers, and health-care managers.

**Figure 1:**
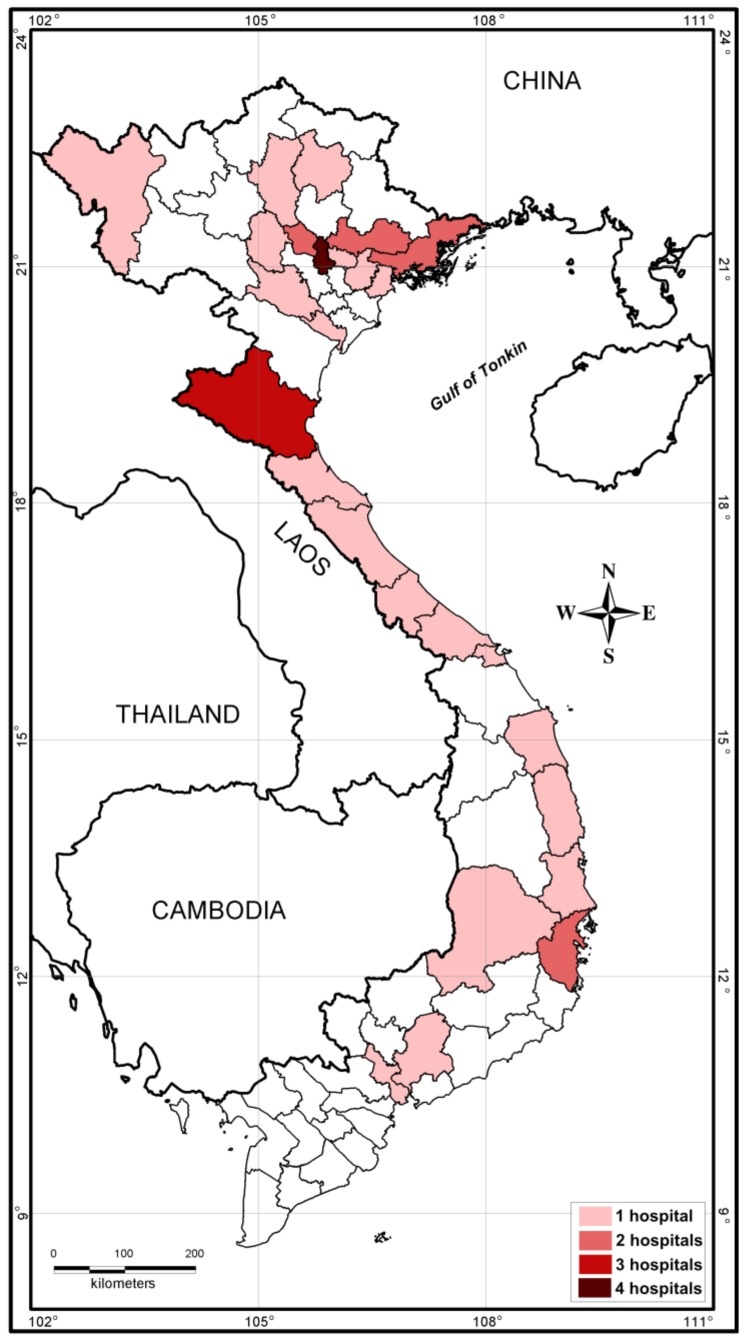
Location of hospitals taking part in the RENOMAB project and reporting culture-confirmed melioidosis cases. All of the *B. pseudomallei* strains were sent to the reference laboratory at the Institute of Microbiology and Biotechnology, Vietnam National University, Hanoi. *B. pseudomallei* identification of bacterial strains was confirmed by using either *recA* sequence analysis or *B. pseudomallei*-specific TTSS1 real-time PCR assay [28]. Border lines of provinces or cities are shown. The color indicates the number of hospitals within the province or city that reported cases of melioidosis. Geographic map was constructed by the MapInfo 7.8 (MapInfo, Troy, NY, USA).

Active melioidosis case-finding reports were presented at national scientific conferences for infectious and respiratory diseases [47,48]. Scientific papers on melioidosis have started to appear in national journals [49]. The existence of the disease was also broadcasted in a wide range of public media such as hospital web portals, newspapers and television programs. A private Facebook group named Research Network on Melioidosis in Vietnam (Hoi Nghien cuu Melioidosis tai Vietnam) was created and consists of approximately 600 members from the healthcare system interested in melioidosis diagnostics. At the time of writing, 36 hospitals in 26 provinces and cities reported culture-confirmed melioidosis cases (Figure 1). Of these, 28 hospitals detected the first cases of the disease after joining case-finding activities within RENOMAB. This project has shown that melioidosis is widely distributed throughout the country with a potential area of high endemicity in North Central Vietnam [28]. The organization of the 9th World Melioidosis Congress in 2019 in Hanoi will be a golden opportunity to further raise awareness for melioidosis and to promote research on different aspects of epidemiology, diagnosis, and treatment in Vietnam.

## 6. Current Recommendations for Diagnosis and Treatment and for Reporting and Prevention

### 6.1. Diagnosis and Treatment

Currently, there are no official guidelines for diagnosis and treatment of melioidosis issued by the Vietnamese Ministry of Health, and *B. pseudomallei* has not been yet on the list of any national surveillance programs for infectious agents. The current recommendations that have been distributed via seminars, workshops, and national conferences, are based on studies and guidelines from the international literature.

In well-equipped laboratories commercially-available biochemical tests and automated identification systems are routinely used. However, even under such circumstances, a lack of awareness and of additional diagnostic tools can lead to unreliable diagnoses, since misidentification of *B. pseudomallei* has been well documented for such systems [50]. In remote areas where laboratory facilities and resources for consumables are still limited, the identification of bacterial pathogens mostly relies on some basic microbiological tests, and the identification of Gram-negative non-fermenting bacterial species is even more challenging. We therefore recently introduced a simple laboratory algorithm for identification of *B. pseudomallei* from clinical specimens under such resource-constrained conditions. The algorithm makes use of the inherent resistance of *B. pseudomallei* to gentamicin and colistin and the susceptibility to amoxicillin-clavulanic acid [28].

Although the sensitivity and specificity of this algorithm have not been validated yet, compared to more sophisticated identification procedures including selective media such as Ashdown’s agar for non-sterile sites, it proved to be an effective and inexpensive procedure leading to the diagnosis of a significant number of melioidosis patients at provincial general hospitals in North Central Vietnam in a short period of time after introduction [28]. We therefore currently recommend the implementation of this simple algorithm in clinical laboratories for a presumptive diagnosis of *B. pseudomallei* from clinical materials. Confirmation of the identification can be obtained in reference laboratories using specific type three secretion system 1 (TTSS1) real-time PCR assays and sequencing of the *recA* gene as reliable target [28].

### 6.2. Surveillance and Prevention

Melioidosis is not a notifiable disease in Vietnam. There is neither a formal surveillance system for human nor for animal melioidosis in Vietnam. There are also no official recommendations on prevention. The limited epidemiological data available in Vietnam indicate that rice farmers are at a particular risk, similar to other parts of Southeast Asia. At present, it seems plausible to primarily target the rice farming population in terms of better diagnostics and evaluation of possible preventive measures. The majority of them are living in remote areas where the primary healthcare system of the country does not cover microbiological investigations. It is therefore most likely that fatal cases of melioidosis in remote areas are still grossly under recognized.

## 7. Current and Future Challenges

Although some progress has recently been made in Vietnam to increase awareness of melioidosis and to enhance laboratory skills for identification of *B. pseudomallei* from clinical specimens, there are still significant efforts needed to further improve capacity building in different regions of Vietnam.

Apart from further improving the identification capacity in the laboratories via diagnostic workshops and scientific conferences it will be crucial to generally increase the number of clinical specimens—such as urine, throat swabs, sputum, pus, etc.—to be sent to the laboratory for microbiological investigations. Since bacteremic melioidosis is a common clinical presentation and also associated with a high case fatality rate, increasing the number of blood cultures in patients with prolonged fever has a high priority. There are financial barriers and insurance issues that hinder an appropriate use of blood cultures in the diagnosis not only for melioidosis, but also for other systemic infections, which need to be addressed. Epidemiological studies are needed to determine the true incidence and prevalence of melioidosis in the various regions. The same is true for the veterinary field, where information on melioidosis is still very scarce.

In parallel to capacity building in the clinical laboratory, studies on the environmental distribution of *B*. *pseudomallei* in Vietnam will be important to define risk areas for the indigenous population more precisely. This information can be used to further target clinical microbiology activities. In addition, serological screening of the indigenous population using newly-developed devices might help to detect exposure to *B*. *pseudomallei* and to identify possible endemic hot spots.

## Figures and Tables

**Table 1:** Number of indigenous Vietnamese patients with melioidosis and the respective outcome reported over time in medical journals.

Year of Reporting	No. of Patient	No. of Recoveries ^a^	No. of Deaths	No. of Unknown Outcome	Reference
1926	1	0	1	0	[3]
1949	9	5	0	4	[6]
1958	1	0	1	0	[10]
1967	1	1	0	0	[11]
1995	7	1	6	0	[24]
1990	1	1	0	0	[25]
1999	9	0	0	9	[26]
2008	55	32	17	6	[27]
2018	70	30	18	22	[28]

^a^ Recovery during the study.
